# The actin capping protein in *Aspergillus nidulans* enhances dynein function without significantly affecting Arp1 filament assembly

**DOI:** 10.1038/s41598-018-29818-4

**Published:** 2018-07-30

**Authors:** Jun Zhang, Rongde Qiu, Xin Xiang

**Affiliations:** Department of Biochemistry and Molecular Biology, The Uniformed Services University of the Health Sciences- F. Edward Hébert School of Medicine, Bethesda, Maryland 20814 USA

## Abstract

The minus-end-directed microtubule motor cytoplasmic dynein requires the dynactin complex for *in vivo* functions. The backbone of the vertebrate dynactin complex is the Arp1 (actin-related protein 1) mini-filament whose barbed end binds to the heterodimeric actin capping protein. However, it is unclear whether the capping protein is a dynactin component in lower eukaryotic organisms, especially because it does not appear to be a component of the budding yeast dynactin complex. Here our biochemical data show that the capping protein is a component of the dynactin complex in the filamentous fungus *Aspergillus nidulans*. Moreover, deletion of the gene encoding capping protein alpha (*capA*) results in a defect in both nuclear distribution and early-endosome transport, two dynein-mediated processes. However, the defect in either process is less severe than that exhibited by a dynein heavy chain mutant or the ∆p25 mutant of dynactin. In addition, loss of capping protein does not significantly affect the assembly of the dynactin Arp1 filament or the formation of the dynein-dynactin-∆C-HookA (Hook in *A*. *nidulans*) complex. These results suggest that fungal capping protein is not important for Arp1 filament assembly but its presence is required for enhancing dynein function *in vivo*.

## Introduction

**Figure 1:**
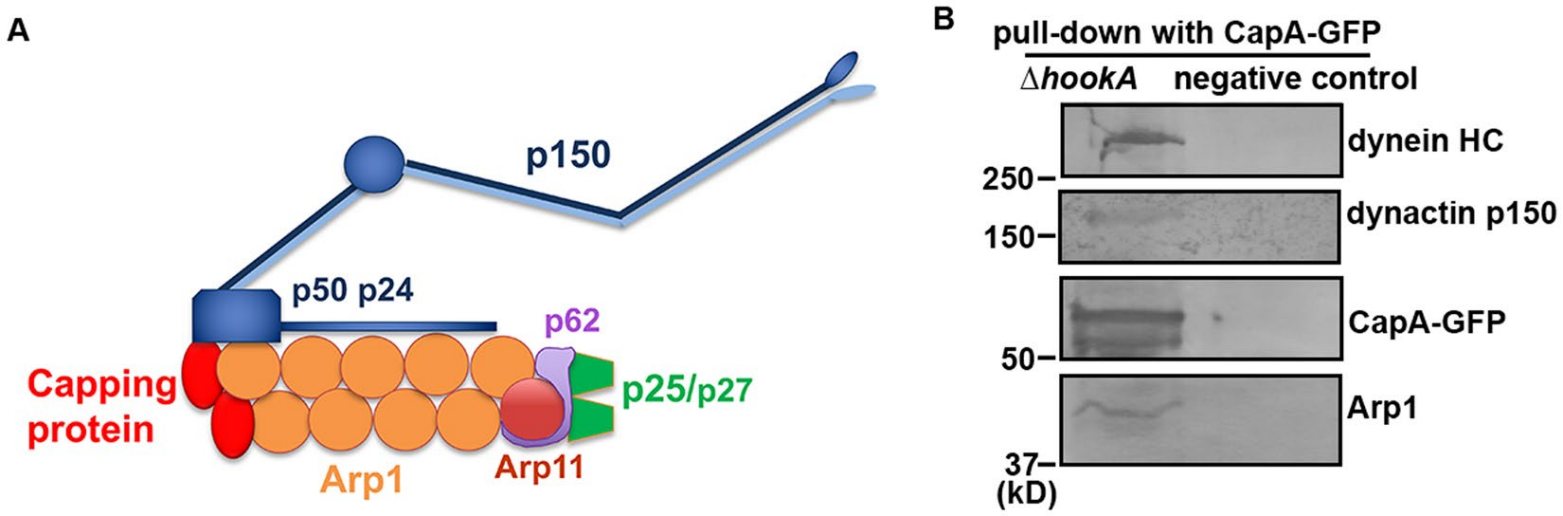
Components of the dynactin complex are pulled down with CapA-GFP. (**A**) A schematic representation of the dynactin complex. Conventional actin was not depicted as we do not have evidence from our pull-down experiments that conventional actin is a component of the *A*. *nidulans* dynactin complex. (**B**) Western blots showing that dynactin p150, Arp1 and the dynein HC were pulled down with CapA-GFP. A strain without any GFP tag was used as a negative control. Cropped pieces with black outlines indicate blots probed by different antibodies against the indicated proteins (see Supplemental Fig. 5 for the original blots). The antibody against GFP (from Clontech) has been used previously^29^. The affinity-purified antibodies against dynein HC, dynactin p150 and Arp1 have been described and used previously^6,40^.

Cytoplasmic dynein, a minus-end-directed microtubule motor, transports a variety of membranous organelles, proteins and mRNAs, and almost all the *in vivo* functions of cytoplasmic dynein require the dynactin complex^1^. The backbone of the vertebrate dynactin complex is the Arp1 (actin-related protein 1) mini-filament of about 37 nm whose pointed end is occupied by pointed-end sub-complex including Arp11, p62, p25 and p27 (Fig. 1A)^2–5^. Studies in fungi and higher eukaryotic cells have suggested that Arp11 and p62 are important for the integrity of the Arp1 filament^6,7^. The peripheral subunits p25 and p27 are not essential for dynactin complex assembly or function but are involved in targeting dynein-dynactin to various cargoes^7–10^. The barbed end of the Arp1 filament in the vertebrate dynactin complex is occupied by the actin capping protein, which is an evolutionarily conserved heterodimer of alpha and beta subunits^2,11^. Capping protein binds a barbed end of an actin filament to block the addition of actin monomers to the end, thereby preventing filament elongation, and it also stops loss of actin monomers from the end^11^. Functional studies of capping protein have been done in different cell types especially in the budding yeast *Saccharomyces cerevisiae*^12–15^. *S*. *cerevisiae* is an excellent genetic system for studying dynein-mediated positioning of mitotic spindles^16–18^, but as dynein is only critical for spindle positioning, several vertebrate components of dynactin are missing in *S*. *cerevisiae*. For example, genes encoding the Arp1 pointed end proteins p62, p25 and p27 are not found in *S*. *cerevisiae* genome^3,19^. The actin capping protein is clearly present in *S*. *cerevisiae*, but interestingly, biochemical studies have found no evidence that the actin capping protein is a component of the *S*. *cerevisiae* dynactin complex^19^. In addition, loss of the capping protein does not significantly affect dynein-mediated spindle positioning, arguing against a critical role of the capping protein in yeast dynein function^19^.

The filamentous fungus *Aspergillus nidulans* is an established model organism for dissecting the functions of dynein and dynactin components. In *A*. *nidulans*, dynein is required for nuclear distribution along hyphae, and mutants defective in dynein function show an abnormal cluster of nuclei in the spore head of a germ tube^18,20^. Besides nuclear distribution, *A*. *nidulans* dynein is also critical for the transport of various cargoes, including the early endosomes and cargoes that move by hitchhiking on early endosomes^21–24^. Early endosomes are transported bi-directionally by kinesin-3 and dynein in filamentous fungi as first shown in *Ustilago maydis*^25,26^. In *A*. *nidulans* mutants that are defective in dynein-mediated early-endosome movement, for example, the deletion mutant of dynactin p25, early endosomes accumulate abnormally at the hyphal tip where the microtubule plus ends are located^9^. It has been shown in both *A*. *nidulans* and *U*. *maydis* that proteins in the Fts-Hook-Fhip (FHF) complex link the dynein-dynactin complexes to the early endosome cargo^27–29^. Consistent with a previously identified role of dynactin p25 in dynein-cargo interaction, we showed that *A*. *nidulans* p25 is required for the physical interaction between HookA (Hook in *A*. *nidulans*) and dynein-dynactin^9,29^. However, while the actin capping protein is a component of the vertebrate dynactin complex that locates at the barbed end of the Arp1 filament^2,3,5,30,31^, it has never been shown to be a dynactin component in *A*. *nidulans* or any other low eukaryotic organisms.

In this work, our biochemical data show that the actin capping protein is a component of the *A*. *nidulans* dynactin complex. Importantly, loss of capping protein results in partial defects in both nuclear distribution and early-endosome transport, two dynein-mediated processes. However, the defect in nuclear distribution or early-endosome movement is much less severe than that exhibited by a dynein heavy-chain mutant or the ∆p25 mutant, respectively. Interestingly, results of our biochemical pull-down assays suggest that loss of the capping protein does not affect dynactin complex integrity in an obvious way. These results suggest that capping protein in the fungal dynactin complex is not essential for dynactin complex assembly but is required for the optimal dynein function *in vivo*.

## Results

### Capping protein is a component of the dynactin complex in *A*. *nidulans*

In *A*. *nidulans*, the capping protein alpha (CapA, 273 aa) is encoded by the gene An2126 on chromosome VII, and the capping protein beta (CapB, 266 aa) is encoded by the gene An0290 on chromosome VIII. Both CapA and CapB show obvious sequence homology with the yeast and human capping protein alpha and beta respectively (Supplemental Figures S1 and S2). We noticed that the predicted open reading frame of CapA in the Aspergillus genome database (AspGD) contains a longer N-terminal region with an extra 96 amino acid stretch that shows no homology with the yeast and human cap alpha proteins. Based on the open reading frame prediction of CapA homologs in *Aspergillus fumigatus* and *Aspergillus oryzae*, we think that this must be due to a mistake in the prediction of CapA start codon and that CapA should start with amino acids MASTVEFA as we show in the alignment (Supplemental Figure S1).

To test whether capping protein is a component of the dynactin complex in *A*. *nidulans*, we performed a protein pull-down experiment using GFP-antibody-conjugated beads and a strain containing the p25-GFP fusion protein^9^. To minimize the pull-down of proteins that associate with dynein-dynactin indirectly via binding to dynein cargoes such as the early endosomes, we introduced the ∆C-HookA-S allele into the p25-GFP strain background by genetic crossing. The C-termini of Hook proteins are important for cargo binding and interaction with FTS and FHIP, and HookA’s C-terminus binds early endosome via FtsA and FhipA whose mammalian homolog FHIP interacts directly with the early endosome marker Rab5^27–29,32–35^. As expected, our proteomic analysis of the pulled-down proteins from the ∆C-HookA-S strain shows that while HookA was pulled down with p25-GFP, neither FtsA (FTS homolog) nor FhipA was pulled down. In addition, kinesin-3 (UncA) was not pulled down, consistent with its association with early endosomes^26,36,37^. In contrast, Kinesin-1 (KinA) was pulled down, suggesting its interaction with dynein-dynactin independent of early endosomes. Importantly, both CapA and CapB were pulled down together with other dynein-dynactin components (Table 1), and none of these proteins were pulled down in our negative control using a wild type strain without any GFP tag (Table 1).

**Table 1:** Proteomic analysis of proteins pulled down with p25-GFP in the ∆C-HookA-S background.

	p25-GFP/∆C-HookA-S	Negative control
CapA (An2126, 273aa)	5	0
CapB (An0290, 266aa)	3	0
Arp1 (An1953, 380aa)	9	0
Arp11 (An3185, 557aa)	15	0
p62 (An4917, 637aa)	11	0
p25 (An5022, 202aa)	3	0
p150 (An6323, 1342aa)	55	0
p50 (An3589, 467aa)	24	0
p24 (An12001, 235aa)	2	0
Dynein HC (An0118, 4345aa)	133	0
Dynein IC (An1454, 689aa)	20	0
Dynein LIC (An4664, 505aa)	19	0
Dynein LC8 (An0420, 94aa)	8	0
Dynein TctexA (An1333, 141aa)	2	0
NudF/Lis1 (An6197, 444aa)	11	0
NudE (An6125, 586aa)	18	0
HookA (An5126, 638aa)	7	0
FtsA (An0883, 328aa)	0	0
FhipA (An10801,843aa)	0	0
Kinesin-1 KinA (An5343, 927aa)	9	0
Kinesin-3 UncA (An7547, 1630aa)	0	0
Myosin V (An8862, 1569aa)	0	0

Protein names and the number of unique peptides detected are listed. The negative control is a strain without any GFP tag, and the protein extracts of the two strains are processed the same way in the experiment (see Supplementary Dataset 1 for all original data).

To verify that the capping protein is associated with the dynactin complex in *A*. *nidulans*, we constructed a strain containing the *capA*-GFP allele at the *capA* locus and examined if components of the dynactin complex are pulled down with CapA-GFP. The strain containing the CapA-GFP fusion forms a colony that is slightly smaller than that of a wild-type strain but bigger than the CapA-deletion mutant (Supplemental Fig. 3), indicating that CapA-GFP is partially but not fully functional. CapA-GFP localizes to actin patches as suggested by the co-localization of CapA-GFP with a known actin patch-associated protein AbpA (Abp1 homolog) (Supplemental Fig. 4)^38,39^. We then used the CapA-GFP strain for the pull-down experiment using GFP-antibody-conjugated beads. To eliminate the pull-down of early endosomes, the ∆*hookA* allele was introduced into the CapA-GFP strain background via genetic crossing. In our proteomic analysis on proteins pulled down by the GFP-antibody, we found not only CapA and CapB but also p150 dynactin (52 unique peptides) along with all other dynactin components as well as dynein heavy chain (32 unique peptides) (see Supplementary Dataset 2 for the original data). We confirmed these interactions by western blot analysis of the proteins pulled-down with CapA-GFP, using affinity-purified antibodies against p150 dynactin, Arp1 and dynein heavy chain (HC)^6,40^ (Fig. 1B and Supplemental Figure 5). These results further support the notion that the capping protein is a component of the *A*. *nidulans* dynactin complex.

### The ∆*capA* mutant is partially defective in dynein-mediated nuclear distribution

**Figure 2:**
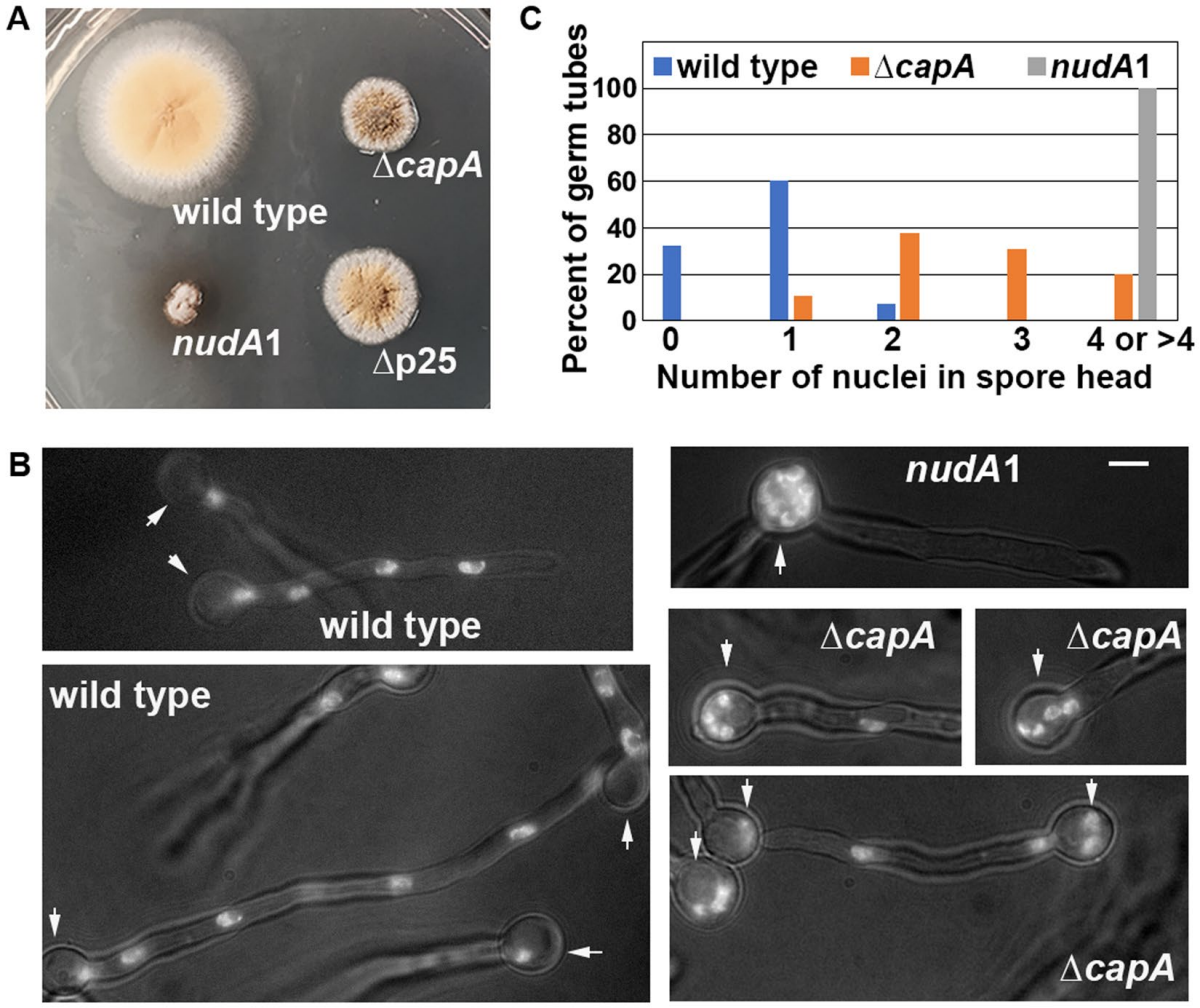
Colony and nuclear-distribution phenotypes of the ∆*capA* mutant. (**A**) Colony phenotypes of the ∆*capA* mutant and control strains including wild type, the *nudA*1 mutant and ∆p25 mutant. The plate was incubated at 37 °C for 2 days. (**B**) Images showing the nuclear distribution phenotype of the ∆*capA* mutant in comparison with that of wild type or the *nudA*1 mutant. Cells were grown at 37 °C for ~8 hours in MM + glucose medium. Bar, 5 μm. (**C**) A quantitative analysis on the percentage of germ tubes containing 0, 1, 2, 3 or ≥4 nuclei in the spore head. In the wild-type control strain, 60.3% of the germ tubes contain one nucleus, 32.4% contain no nucleus and 7.3% contain two nuclei in the spore head (n = 68). In the ∆*capA* mutant, 10.7% contain one nucleus, 38.1% contain two nuclei, 31% contain three nuclei, 17.8% contain four nuclei and 2.4% contain five nuclei in the spore head (n = 84). The mean ranks of these two sets of data are significantly different at *p* = 0.05 (the actual *p*-value is smaller than 0.000000000000001, two-tailed) based on a nonparametric test that assumes no information about the distribution (unpaired, Mann-Whitney test, Prism 7 for Mac OS X, version 7.0c, 2017). In the *nudA*1 mutant, 100% of the germ tubes (n = 50) show a cluster of 4–8 nuclei in the spore head, and we express the number as “4 or >4” because the exact number of nuclei in the cluster is hard be determined accurately.

To study the function of the capping protein, we deleted the whole *capA* open reading frame and a ~420-bp region before the start codon (note that there is no other gene within this region) (Supplemental Fig. 6). In *A*. *nidulans*, dynein is responsible for even distribution of multiple nuclei in hyphae, and nuclear distribution (*nud*) mutations that eliminate dynein or dynactin function cause a small-colony phenotype^20,41^. The ∆*capA* mutant formed a colony obviously smaller and more compact than a wild-type colony, and it is also smaller than the colony of the ∆p25 mutant defective in dynein-mediated early endosome transport but not in nuclear distribution (Fig. 2A). However, the colony of the ∆*capA* mutant is bigger than a typical nud colony formed by the *nudA*1 mutant at its restrictive temperature, suggesting that loss of CapA does not completely abolish dynein function (Fig. 2A). We have also made a *capB* (encoding capping protein beta) deletion mutant in *A*. *nidulans*, which shows the same colony phenotype as ∆*capA* (Supplemental Fig. 3), fully consistent with the well-established notion that heterodimer formation is essential for capping protein function^42^. In this work, we mainly focused on ∆*capA* for analyzing capping protein function.

To determine if the ∆*capA* mutant exhibits any defect in dynein-mediated nuclear distribution, we introduced the previously used GFP-labeled Histone H1 fusion^36,43^ into the mutant and compared its nuclear distribution pattern with those of wild type and a temperature-sensitive *nudA*1 dynein heavy-chain (HC) mutant. Nuclei are evenly distributed in a wild-type germ tube upon spore germination, but in the *nudA*1 mutant grown at its restrictive temperature of 37 °C for ~8 hours in MM + glucose medium, multiple nuclei (4 or >4) are clustered in the spore head as they fail to migrate out into the germ tube (Fig. 2B)^18,20^. Under the same conditions, the ∆*capA* mutant clearly exhibited a defect in nuclear distribution as more germ tubes contain 2 or more nuclei in the spore head, but the defect was much less severe than that exhibited by the *nudA*1 mutant (Fig. 2B,C). Thus, the capping protein is important but not essential for dynein-mediated nuclear distribution.

During this analysis, we also observed nuclear distribution in wild type and the ∆*capA* mutant grown under several other conditions. Specifically, cells were grown at 32 °C in MM + glycerol or MM + glucose medium or at 37 °C in MM + glycerol medium (Supplemental Fig. 6A–C). We found that under all conditions, the ∆*capA* mutant exhibited a partial defect in nuclear distribution, although this defect seems more obvious at 37 °C in general as evidenced by a higher percentage of germ tubes containing 3–4 nuclei in the spore head (Fig. 2C and Supplemental Fig. 7A–C).

**Figure 3:**
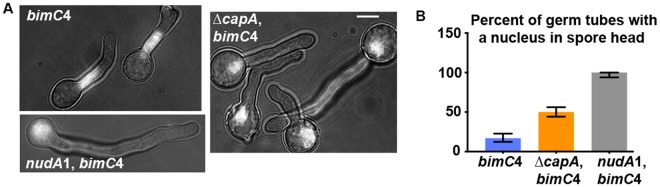
The nuclear-distribution phenotype of the ∆*capA* mutant in the *bimC*4 background. (**A**) Images showing the position of the single nucleus in the *bimC*4 mutant, the *nudA*1, *bimC*4 double mutant and the ∆*capA*, *bimC*4 double mutant. Cells were grown at 42 °C or ~8 hours in MM + glucose medium. Bar, 5 μm. (**B**) A quantitative analysis on the percentage of germ tubes with the single nucleus in the spore head. The percentage values are 16.9% for the *bimC*4 single mutant (n = 201), 50.2% for the ∆*capA*, *bimC*4 double mutant (n = 263) and 100% for the *nudA*1, *bimC*4 double mutant (n = 60). Error bars represent the 95% confidence interval values generated by Prism 7.

The presence of multiple nuclei in the spore head could indicate a defect either in nuclear migration or nuclear separation after division as the cluster of connected nuclei may be too big to migrate into the narrow germ tube^20,44^. To confirm that loss of CapA indeed causes a defect in nuclear migration we introduced via genetic crossing the temperature-sensitive *bimC*4 (Kinesin-5, a mitotic kinesin) mutation into the strains of wild type, ∆*capA* and *nudA*1 together with GFP-labeled histone H1. In the *bimC*4 mutant grown at its restrictive temperature of 42 °C for ~8 hours, the loss of kinesin-5 function leads to the presence of a big polyploid nucleus due to a failure in bi-polar spindle formation and nuclear division but not in DNA replication^45^. The single nucleus can migrate out of the spore head in most of the *bimC*4 single mutant germ tubes but not in any of the *nudA*1, *bimC*4 double mutant germ tubes (Fig. 3A), indicating that dynein is required for the movement of the nucleus out of the spore head. In the ∆*capA*, *bimC4* double mutant, nuclear migration out of the spore head is obviously defective (Fig. 3A), but still happens in ~50% of the germ tubes (n = 263) (Fig. 3B). Thus, the capping protein enhances dynein-mediated nuclear migration but is not essential for this process.

### The ∆*capA* mutant is partially defective in dynein-mediated early-endosome transport

**Figure 4:**
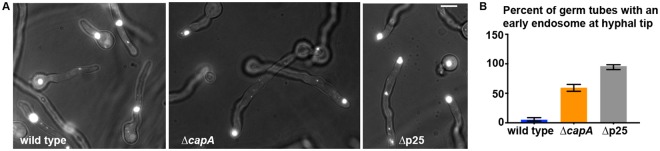
Early endosome-distribution phenotype of the ∆*capA* mutant. (**A**) Images showing large early endosomes labeled by overexpressed GFP-RabA in wild type and the ∆*capA* mutant. Cells were grown at 37 °C for ~8 hours in MM + glucose medium. The ∆p25 mutant defective in the dynein-early endosome interaction was used as a control. Bar, 5 μm. (**B**) A quantitative analysis on the percentage of germ tubes with a large early endosome located at the hyphal tip. The percentage values are 5.1% for wild type (n = 234), 59.4% for the ∆*capA* mutant (n = 271), and 96% for the ∆p25 mutant (n = 100). Error bars represent the 95% confidence interval values generated by Prism 7.

Beside nuclear distribution, another excellent readout of dynein function in *A*. *nidulans* is the distribution of early endosomes. As microtubules between the hyphal tip and the most tip-proximal nucleus have their plus ends facing the hyphal tip^36,46–49^, a defect in dynein-mediated transport results in an abnormal accumulation of early endosomes at the hyphal tip^25^. To determine if the capping protein is important for early-endosome distribution, we introduced into the ∆*capA* mutant a routinely used early endosome marker, the *alcA*-promoter-driven mCherry-RabA fusion^9,36,50^. We incubated the cells at 32 °C in MM + glycerol medium that allows expression of the marker. In contrast to the dramatic buildup of early endosomes at hyphal tips in the ∆p25 mutant^9^, there was very limited or no accumulation of early endosomes at the hyphal tips of the ∆*capA* mutant. We then sought to observe early endosomes at 37 °C on MM + glucose medium, as our analysis on nuclear distribution suggested that the phenotype of the ∆*capA* mutant could be more pronounced under this condition. To do that, we used strains containing the GFP-RabA fusion whose expression is driven by the *gpdA*^mini^ promoter^9^. The *gpdA*^mini^ promoter results in high expression of GFP-RabA, causing early endosomes to undergo RabA-mediated fusion^51^. Thus, the strains carrying overexpressed GFP-RabA contain large early endosomes (Fig. 4A). Upon inoculation of asexual spores on MM + glucose medium for ~8 hours at 37 °C, most germ tubes contain one or more of these large early endosomes. Although the movements of these large early endosomes were much less robust than those of the mCherry-RabA-labeled early endosomes, the relative position of each large early endosome is an excellent readout of dynein function. In wild-type strains, a large early endosome is usually seen in the middle of the germ tube (Fig. 4A). In contrast, it is almost always seen at the hyphal-tip region in the ∆p25 mutant (Fig. 4A), which is defective in recruiting dynein to early endosomes^9^. In the ∆*capA* mutant, about 59.4% the germ tubes have a large early endosome at the hyphal-tip region (n = 271) (Fig. 4A,B), indicating a defect in dynein-mediated transport.

During this analysis, we also observed these large early endosomes under other culture conditions, such as in MM + glycerol medium at 32 °C or 37 °C for overnight. We found that under these conditions, the ∆*capA* mutant also exhibited a partial defect in early-endosome distribution (Supplemental Fig. 8A,B).

In *A*. *nidulans*, dynein and dynactin form comet-like structures near the hyphal tip, representing their accumulation at the dynamic plus ends of microtubules^49,52^. The microtubule plus-end accumulation of dynein is important for early-endosome transport in filamentous fungi^25^. Thus, we introduced the GFP-dynein HC fusion and the dynactin p150-GFP fusion into the ∆*capA* background by genetic crossing and examined their localizations after the cells were grown at 37 °C for ~8 hours in MM + glucose medium. The comets formed by both fusions appeared normal in the absence of CapA (Supplemental Fig. 9).

### Capping protein is not essential either for the assembly of the Arp1 filament or dynein-dynactin’s interaction with ∆C-HookA

**Figure 5:**
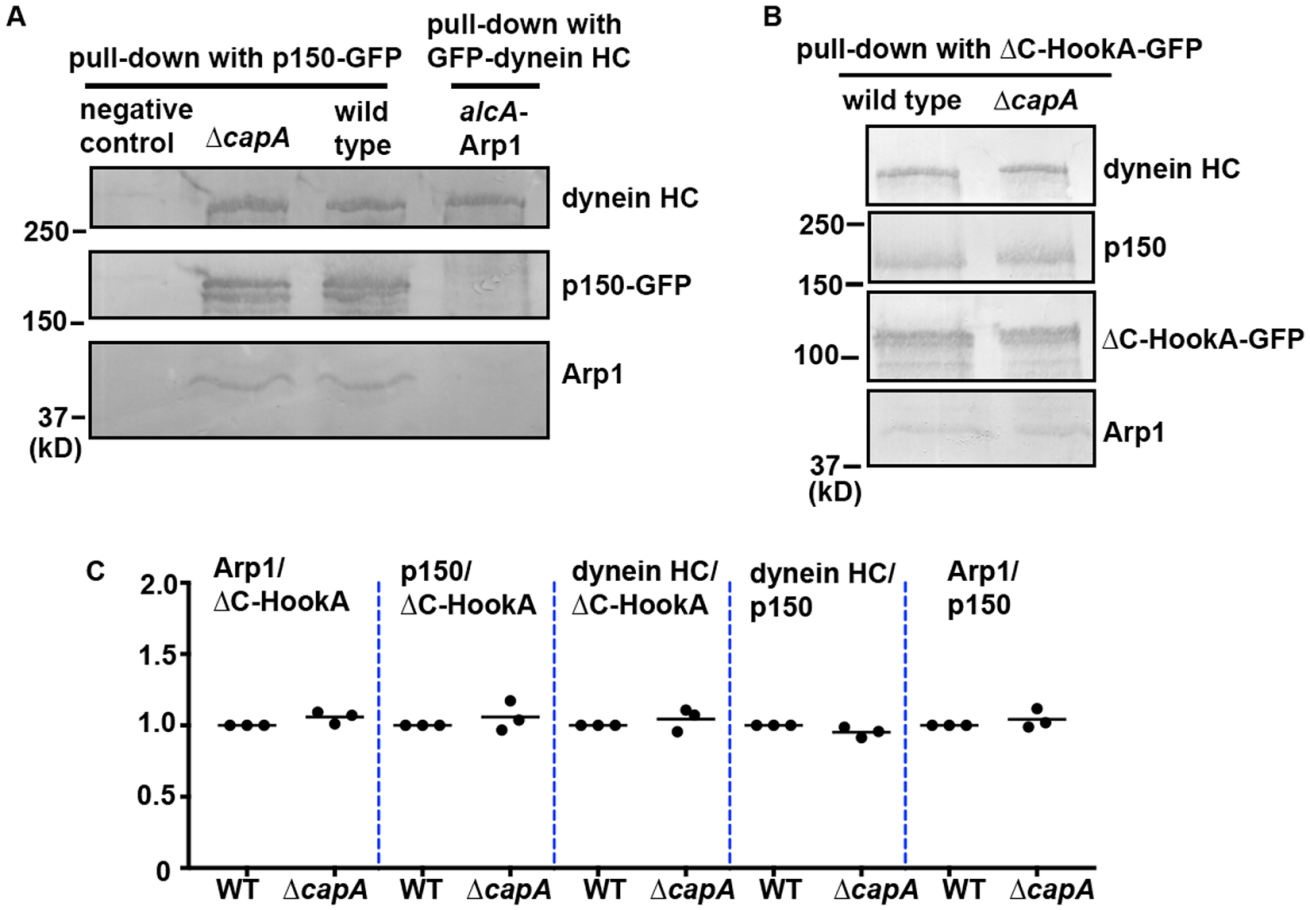
Western analyses of the dynactin complex in the ∆*capA* mutant. (**A**) Western blots showing that normal amounts of dynein HC and Arp1 are pulled down with p150-GFP in the ∆*capA* mutant extract. Cells were cultured for overnight at 37 °C in MM + glucose medium. A strain without any GFP tag was used as a negative control. Another control is the strain containing GFP-dynein HC in the *alcA*-Arp1 background, in which the expression of Arp1 was repressed by glucose and the stability of p150 is decreased^6^. Cropped pieces with black outlines indicate blots probed by different antibodies against the indicated proteins (see Supplemental Fig. 10 for the original blots). (**B**) Western blots showing that dynein HC, dynactin p150 and Arp1 are pulled down with ∆C-HookA-GFP in the ∆*capA* mutant extract. The antibody against GFP (from Clontech) has been used previously^29^. The affinity-purified antibodies against dynein HC, dynactin p150 and Arp1 have been described and used previously^6,40^. Cropped pieces with black outlines indicate blots probed by different antibodies against the indicated proteins (see Supplemental Fig. 11 for the original blots). (**C**) A quantitative analysis on the ratio of pulled-down p150, Arp1 or dynein HC to ∆C-HookA-GFP as well as the ratio of pulled-down dynein HC or Arp1 to p150. The values were generated from western analyses (shown in B) of three independent pull-down experiments. The wild-type values are set as 1. Scatter plots with mean values were generated by using Prism 7. For all the ratios, there is no significant difference between wild-type and ∆*capA* at the 95% confidence level based on nonparametric tests without assuming any information on the distribution (*p* = 0.1 for Arp1/∆C-HookA, *p* = 0.7 for p150/∆C-HookA, *p* = 0.7 for dynein HC/∆C-HookA, p = 0.1 for dynein HC/p150 and p = 0.7 for Arp1/p150, two-tailed) (unpaired, Mann-Whitney test, Prism 7).

To determine the integrity of the dynactin complex upon loss of the capping protein, we introduced the dynactin p150-GFP fusion into the ∆*capA* mutant by genetic crossing and performed biochemical pull-down experiments using cells grown for overnight at 37 °C. Our proteomic analysis of the pull-down materials from the ∆*capA* background shows that all dynactin components except for CapA or CapB are still present upon loss of CapA (Supplemental Table 1). The amounts of dynein HC and Arp1 pulled down with p150-GFP appeared normal based on western blot analysis (Fig. 5A and Supplemental Fig. 10).

We next determined whether the capping protein affects the interaction of dynein-dynactin with the dynein-dynactin-binding portion of HookA (the early endosomal adapter for dynein), ∆C-HookA^29^. Specifically, we performed biochemical pull-down assays using strains containing ∆C-HookA-GFP^29^ after the cells were cultured at 37 °C for overnight. We found similar association of p150 and Arp1 of the dynactin complex as well as dynein HC with ∆C-HookA-GFP in the presence or absence of CapA, suggesting that loss of the capping protein does not significantly affect the formation of the dynein-dynactin-∆C-HookA super-complex (Fig. 5B,C and Supplemental Fig. 11). Recently, dynein cargo adapters missing the C-terminal cargo-binding region have been shown to activate the processive movement of dynein *in vitro*^53–56^. In addition, cryo-EM-based structural studies on the dynein-dynactin-cargo adapter super-complex found that one dynactin complex is able to recruit two dynein dimer complexes^30,31^. Interestingly, one tail of the second dynein dimer is located in between the capping protein and its adjacent Arp1 subunit (Arp1B)^30,31^. Since this was observed in the dynein-dynactin-Hook3 (without the C-terminus) complex, we quantitated the ratio of dynein HC to p150 pulled down with ∆C-HookA-GFP. We detected no significant difference between samples with or without CapA (Fig. 5C). In addition, the ratio of Arp1 to p150 was not significantly affected by the loss of CapA either (Fig. 5C). Thus, although we initially hypothesized that without the barbed-end capping protein the Arp1 filaments may be longer, our current data do not provide any support for this idea. Rather, our results suggest that the capping protein is neither essential for the assembly of the Arp1 filament within the dynactin complex nor important for the dynein-dynactin-∆C-HookA interaction.

### Capping protein affects both the frequency and velocity of dynein-mediated early endosome movement

Although CapA does not significantly affect the integrity of the dynactin complex or the dynein-dynactin-∆C-HookA interaction, it is possible that its presence may enhance dynein motility or force production when dynein carries its cargo *in vivo*. In filamentous fungi, kinesin-3 moves early endosome toward the hyphal tip, and when dynein binds to the same early endosome, it overpowers kinesin-3 and switches the direction of movement^36,57^. To examine CapA function in more detail, we analyzed both dynein- and kinesin-mediated early endosome movements in strains containing the TagGFP2-RabA fusion under the control of the endogenous promoter of *rabA*^36^. Compared to the strains containing overexpressed GFP-RabA fusion, these strains contain early endosomes that are much smaller and the GFP signals are much weaker, but motility of these early endosomes can be detected (Supplemental Movie 1)^36^.

**Figure 6:**
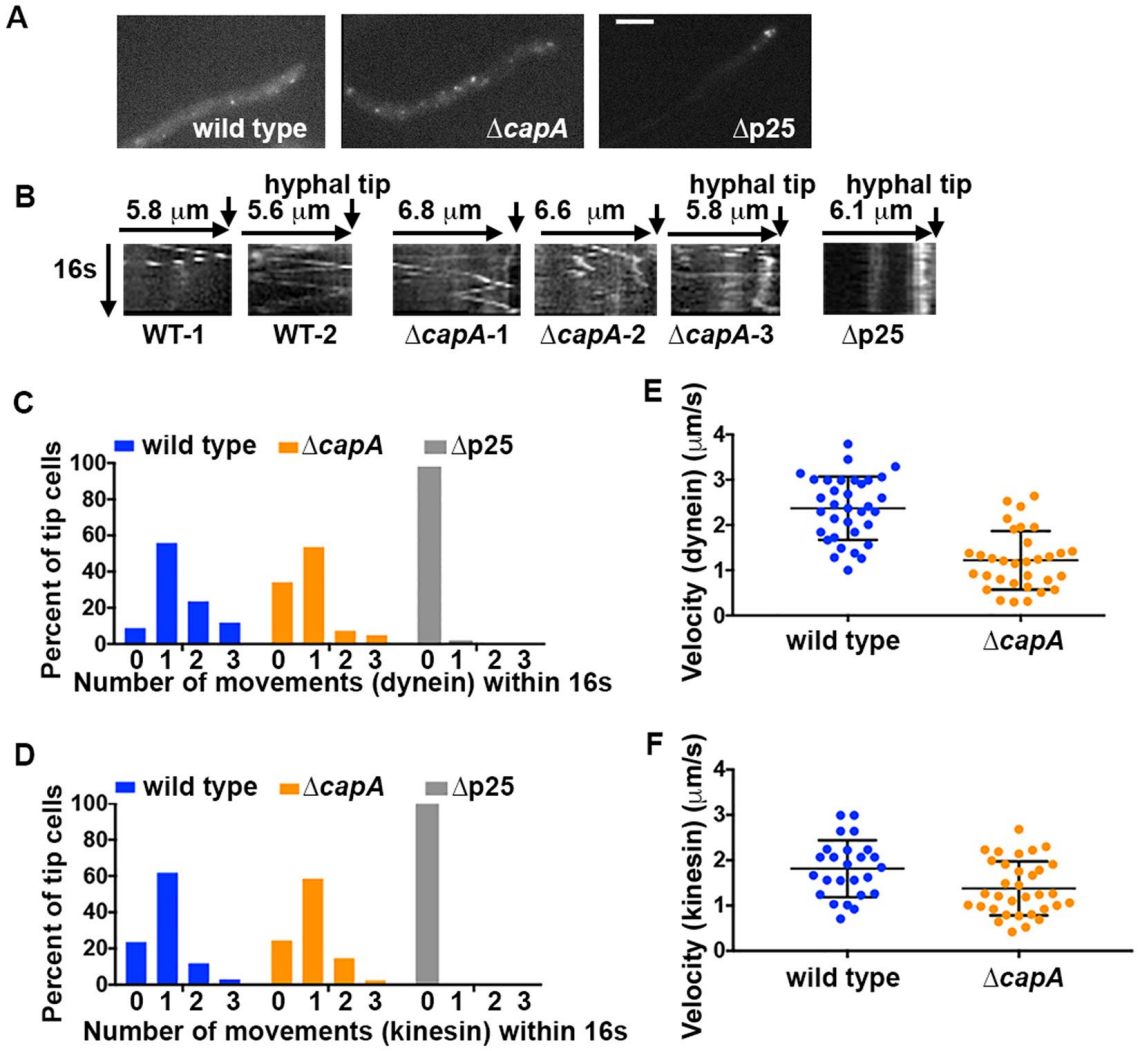
A quantitative analysis of dynein-mediated early endosome movement upon loss of CapA. (**A**) Images showing TagGFP2-RabA-labeled early endosomes in wild type, the ∆*capA* mutant and the ∆p25 mutant. Cells were cultured for overnight at 37 °C in MM + glycerol medium. Bar, 5 μm. (**B**) Kymographs showing early endosome movements. Two kymographs are shown for wild type (WT-1 and WT-2) and three are shown for the ∆*capA* mutant (∆*capA-*1, ∆*capA*-2, and ∆*capA*-3). A ∆p25 kymograph is shown as a control as there is no diagonal lines that indicate movements. For each kymograph, position of the hyphal tip is on the right side and indicated by a short arrow (the word “hyphal tip” is on the last kymograph for each strain). (**C**) Percent of hyphal-tip cells (called “tip cells” for simplicity) showing different numbers of dynein-mediated early endosome movement events within 16 seconds (n = 34 for wild type and n = 41 for the ∆*capA* mutant). The mean ranks of the wild type and ∆*capA* numbers of events are significantly different at *p* = 0.05 (*p* = 0.0022, two-tailed). (**D**) Percent of hyphal-tip cells showing different numbers of kinesin-3-mediated early endosome movements within 16 seconds (n = 34 for wild type and n = 41 for the ∆*capA* mutant). The mean ranks of the wild type and ∆*capA* numbers of events are not significantly different at *p* = 0.05 (*p* = 0.962, two-tailed). (**E**) Velocity of dynein-mediated early endosome movement in wild type and the ∆*capA* mutant (n = 34 for wild type and n = 32 for the ∆*capA* mutant). The mean ranks of the two sets of values are significantly different at *p* = 0.05 (*p* = 0.000000008666721, two-tailed). (**F**) Velocity of kinesin-3-mediated early endosome movement in wild type and the ∆*capA* mutant (n = 25 for wild type and n = 33 for the ∆*capA* mutant). The mean ranks of the two sets of values are significantly different at *p* = 0.05 (*p* = 0.013, two-tailed). Scatter plots with mean and SD values were generated by Prism 7. All the statistical analyses were done using nonparametric tests assuming no information about the distribution (unpaired, Mann-Whitney test, Prism 7).

To perform a quantitative analysis on early endosome movement, we grew cells in MM + glycerol medium for overnight at 37 °C and obtained time-lapse series at 37 °C. Under this condition, most hyphal tips of the ∆p25 mutant contained a small but easily noticeable early-endosome accumulation, but this was unobvious or not observed in the ∆*capA* mutant (Fig. 6A, Supplemental Movies 2 and 3). Directional early-endosome movement was nearly abolished in the ∆p25 mutant (Fig. 6B–D). Although this dramatic decrease in dynein-mediated transport was expected since p25 is required for the dynein-early endosome interaction, it was unclear why kinesin-mediated transport was not observed in the ∆p25 mutant^9^. Previous studies suggested that dynein and kinesin bound to the same cargo could activate each other and loss of dynein function decreases the frequency and/or velocity of kinesin-mediated movements^58–61^. This could be one explanation for our observation, although the lack of early endosome-bound dynein is unlikely to cause a complete loss or inactivation of early endosome-bound kinesin-3 as early endosomes still accumulate at the hyphal tip (Fig. 6A,B).

In the ∆*capA* mutant, movements were detected (Fig. 6B). We measured the frequency and velocity of dynein-mediated movement away from the hyphal tip and kinesin-3-mediated movement toward the hyphal tip (Fig. 6B). We found that the frequency of dynein-mediated movements but not that of kinesin-mediated movements is lower in the ∆*capA* mutant than in the wild type (Fig. 6C,D). This is unlikely caused by a defect in dynein-early-endosome interaction because the early endosome-bound HookA appeared to interact with dynein-dynactin normally in the ∆*capA* extract as judged by a pull-down assay, which is in contrast to the loss of interaction in the ∆p25 mutant used as a negative control (Supplemental Fig. 12). Importantly, the velocity of dynein-mediated movements is significantly reduced in the ∆*capA* mutant (Fig. 6E), indicating that dynein activity is compromised upon loss of the capping protein. Interestingly, the velocity of kinesin-mediated movements is also decreased in the ∆*capA* mutant (Fig. 6F). Although the underlying mechanism is unclear, we would like to speculate that although dynein normally overpowers kinesin-3^36,57^, kinesin-3 may have a better chance to overpower dynein in the absence of CapA, but the resulted plus-end-directed movement would be slowed due to dynein-exerted force of opposite direction.

## Discussion

Using *A*. *nidulans*, we show for the first time that the actin capping protein is a component of the dynactin complex in filamentous fungi and plays an important albeit non-essential role in dynein-mediated processes. Since loss of the capping protein produces only a partial defect in dynein-mediated nuclear distribution and early endosome transport, the capping protein is unlikely to play an essential role in dynactin complex assembly. This notion is consistent with our biochemical result first showing that loss of the capping protein causes no obvious defect in the formation of the dynein-dynactin-∆C-HookA super-complex, which requires both dynein and dynactin complexes^29,53^.

Our results are particularly interesting in light of earlier studies on the involvement of yeast or mammalian capping protein in dynein function^19,62^. In the budding yeast, there is no evidence for the capping protein to be a component of the dynactin complex, and the capping protein is not important for dynein-mediated spindle orientation^19^. In contrast, the capping protein seems to be essential for dynein function in spindle orientation in mammalian cells, although its biochemical mechanism of action remains unclear^62^. Because *A*. *nidulans* dynein plays a role in vesicle transport similar to dynein in mammalian cells, our results suggest that the capping protein may have gained its importance in dynein function during evolution.

We would not completely rule out the possibility that the nuclear-distribution and early endosome-movement phenotypes are partially related to a defect in the actin cytoskeleton, but a major contribution from the actin cytoskeleton seems unlikely. In filamentous fungi, dynein is essential for nuclear distribution, and the actin cytoskeleton does not have any significant impact on nuclear distribution, which differs from the situation in budding yeast where the actin cytoskeleton is important for the Kar9 pathway that partially overlaps with the dynein pathway for nuclear migration/spindle orientation^16–18,20,63–69^. In addition, we have found previously that treatment with the actin depolymerizing drug latrunculin does not significantly affect dynein-mediated early endosome movement in *A*. *nidulans*^70^. Thus, we think that the abnormality in nuclear distribution and early endosome transport in the ∆*capA* mutant is mainly caused by a defect in dynein function.

Our biochemical data suggest that the Arp1 filament within the dynactin complex is unlikely to be elongated or shortened significantly upon loss of the capping protein. This is interesting given the known function of capping protein in controlling the length of conventional actin filaments^11^. While future EM-based structure studies need to be done to address whether there are very subtle changes in Arp1 filament, our result suggests the possibility that capping protein may affect dynein function directly. Recently, two cryo-EM studies on the dynein-dynactin-cargo adapter super-complexes indicate that one dynactin complex is able to recruit two dynein complexes and that the binding site for one tail of the second dynein complex is in between the capping protein and its adjacent Arp1 subunit^30,31^. Although our quantitative western analyses on the ∆C-HookA-dynein-dynactin super-complex do not indicate any significant role of capping protein in dynein recruitment to dynactin, it is possible that position of dynein on the Arp1 filament could be altered subtly without the capping protein, preventing dynein from achieving its optimal level of activity^30,31^.

Our quantitative analyses suggest that both the frequency and velocity of dynein mediated-early endosome movements are reduced upon loss of the capping protein. Since the ability of dynein-dynactin to interact with the cargo adapter HookA is not apparently impaired in the ∆*capA* mutant, we speculate that the defect in dynein-mediated early endosome movement is more likely caused by a decrease in the capacity of dynein to overpower kinesin-3. In this study, we found that the velocity but not the frequency of kinesin-3-mediated early endosome transport is reduced in the ∆*capA* mutant. Although we do not fully understand the underlying mechanism, we speculate that while dynein normally overpowers kinesin-3 on the same early endosome^36,57^, its activity is compromised upon loss of the capping protein so that some minus end-directed movements would be prematurely switched to plus end-directed movements with lowered velocity due to competition from dynein.

Combined with previous studies on the dynactin complex, this current study clearly revealed the fact that proteins at the two opposite ends of the Arp1 filament are not equally needed for Arp1 filament assembly within the dynactin complex. While the pointed-end proteins Arp11 and p62 are both required for Arp1 filament assembly^6,7^, the capping protein is not required for Arp1 filament assembly, although its loss appears to partially impair dynein activity. Thus, the pointed-end proteins are much more important than the barbed-end capping protein during the assembly of the Arp1 filament.

## Materials and Methods

### *A*. *nidulans* strains, media and mutagenesis

*A*. *nidulans* strains used in this study are listed in Table 2. The *hhoA*(histone H1)-GFP-*AfriboB* and TagGFP2-*rabA-AfpyrG* fusions^36,43,71^ as well as other fusions are referenced in Table 2 and/or made by standard methods^72^. For live-cell imaging experiments, cells were cultured in MM + glycerol (minimal medium with 1% glycerol) or MM + glucose (minimal medium with 0.1% glucose) liquid medium (with supplements) for ~8 hours or overnight. In *A*. *nidulans*, hyphal growth is more robust at 37 °C than at 32 °C and slightly more robust in MM + glucose than in MM + glycerol. We normally use MM + glycerol medium to culture cells at 32 °C for observing mCherry-RabA-labeled early endosomes because the mCherry-RabA fusion gene is driven by the *alcA* promoter that is repressed by glucose but not by glycerol, and also because the mCherry-RabA signals are good at 32 °C but not at 37 °C. In this work, we used multiple culture conditions to study the phenotype of the ∆*capA* mutant. Specifically, we observed cells containing the GFP-labeled Histone H1 fusion (*hhoA*-GFP) grown at 32 °C and 37 °C in both MM + glycerol and MM + glucose media. We also observed nuclei in the *bimC*4 mutant background at 42 °C in MM + glucose medium because 42 °C is the restrictive temperature of the *bimC*4 mutant. To study early-endosome distribution, we observed cells containing GFP-labeled RabA (driven by the *gpdA*^mini^ promoter) grown at 32 °C and 37 °C in MM + glycerol medium and at 37 °C in MM + glucose medium. We also observed cells containing TagGFP2-labeled RabA at 37 °C in MM + glycerol medium. Solid medium for plates was made with YG + UU or the MM + glucose medium. The YG + UU medium contains yeast extract (5 g/L), glucose (20 g/L), uridine (1.2 g/L) and uracil (1.2 g/L). To isolate proteins for biochemical analyses on the ∆*capA* mutant, cells were grown at 37 °C in MM + glucose or YG rich medium for overnight. For growing strains on plates, either YG rich medium (with agar) or MM + glucose medium (with agar) was used.

**Table 2:** *A*. *nidulans* strains used in this study.

Strain	Genotype	Source
MAD3131	*pyroA*4[*pyroA-gpdA*^mini^::GFP-*RabA*]; *pantoB*100; *yA*2	^9^
MAD1399	*abpA*-mRFP::*AfpyrG*; *pabaA*1, *pyrG*89, *yA*2	^39^
RQ2	GFP-*nudA*^HC^*; argB*2::[argB*-alcAp::mCherry*-RabA];* ∆*nkuA::argB; pyrG*89*; pyroA*4*; yA*2	^76^
RQ54	*argB*2::[argB*-alcAp::mCherry*-RabA];* ∆*nkuA::argB; pyrG*89*; pyroA*4*; wA*2	^76^
TNO2A3	*∆nkuA::argB*; *pyrG*89; *pyroA*4	^75^
XY42	*argB*2::[argB*-alcAp::mCherry*-RabA];* ∆*nkuA::argB; pyrG*89*; pantoB*100*; yA*2	Xuanli Yao
JZ400	∆p25::*AfpyrG; pyroA4*[*pyroA*-*gpdA*^mini^::GFP::*RabA*]; possibly ∆*nkuA::argB; pyrG*89	This study
JZ476	CapA-GFP-*AfpyrG;* ∆*nkuA::argB; pyrG*89*; pyroA*4	This study
JZ500	HookA-GFP-*AfpryG*; *argB*2::[argB*-alcAp::mCherry*-RabA];* ∆*nkuA::argB; pyrG89; pyroA4; wA2*	^29^
JZ528	HookA-GFP-*AfpryG*; ∆p25::*AfpyrG; argB*2::[argB*-alcAp::mCherry*-RabA;* ∆*nkuA::argB; pyrG89*, *yA2*	This study
JZ711	∆*capA*::*AfpyrG*; GFP-*nudA*^HC^*; argB*2::[argB*-alcAp::mCherry*-RabA];* ∆*nkuA::argB; pyrG*89*; pyroA*4*; yA*2	This study
JZ712	∆*capA*::*AfpyrG*; *argB*2::[argB*-alcAp::mCherry*-RabA];* ∆*nkuA::argB; pyrG*89*; pantoB*100*; yA*2	This study
JZ717	∆*capA*::*AfpyrG*; p150^nudM^-GFP*-AfpyrG; argB*2::[argB*-alcAp::mCherry*-RabA];* ∆*nkuA::argB; pyrG*89	This study
JZ768	∆*capA*::*AfpyrG*; *pyroA*4[*pyroA*-*gpdA*^mini^::GFP::*RabA*]; *possibly* ∆*nkuA::argB; pyrG*89*; yA*2	This study
JZ773	CapA-GFP-*AfpyrG; abpA*-mRFP::pyrGAf; *pyrG*89; *yA*2*; possibly* ∆*nkuA::argB*	This study
JZ774	CapA-GFP-*AfpyrG;* ∆*hookA*::Af*pyrG*; ∆*nkuA::argB; pyrG*89*; wA*2	This study
JZ775	∆*capB*::*AfpyrG*; *argB*2::[argB*-alcAp::mCherry*-RabA];* ∆*nkuA::argB; pyrG*89*; pantoB*100*; yA*2	This study
JZ813	∆*capA*::*AfpyrG*; ∆C-HookA-GFP*-AfpyrG*; *argB*2::[argB*-alcAp::mCherry*-RabA];* ∆*nkuA::argB; pyrG89; yA2*	This study
JZ823	HookA-GFP-*AfpryG*; ∆*capA*::*AfpyrG; pyrG89*, *argB*2::[argB*-alcAp::mCherry*-RabA;* ∆*nkuA::argB*	This study
RQ132	p25-GFP-*AfpyrG*; ∆C-HookA-S-*AfpyrG*; *argB*2::[argB*-alcAp::mCherry*-RabA;* possibly ∆*nkuA::argB;* possibly *pyrG89*	This study
XX390	*hhoA*-GFP-*AfriboB*; *pyrG*89; ∆*nkuA::argB; fwA*	This study
XX401	*bimC4; hhoA*-GFP-*AfriboB*; *argB*2::[argB*-alcAp::mCherry*-RabA; pyrG*89; ∆*nkuA::argB; yA2*	This study
XX422	∆*capA*::*AfpyrG*; *bimC4; hhoA*-GFP-*AfriboB*; *argB*2::[argB*-alcAp::mCherry*-RabA; pyrG*89; ∆*nkuA::argB; yA2*	This study
XX406	∆*capA*::*AfpyrG*; *hhoA*-GFP-*AfriboB*; *pyrG*89; ∆*nkuA::argB; yA2*	This study
XX413	[TagGFP2-*rabA-AfpyrG*]; *argB*2::[argB*-alcAp::mCherry*-RabA; yA2;* possibly *pyrG*89; possibly ∆*nkuA::argB*	This study
XX415	∆*capA*::*AfpyrG*; [TagGFP2-*rabA-AfpyrG*]; *argB*2::[argB*-alcAp::mCherry*-RabA; yA2;* possibly *pyrG*89; possibly ∆*nkuA::argB*	This study
XX431	*bimC*4; *nudA*1; *hhoA*-GFP-*AfriboB*	This work
XX432	*nudA*1; *hhoA*-GFP-*AfriboB*	This work
XX461	∆p25::*AfpyrG*; [TagGFP2-*rabA-AfpyrG*]; *yA*2; possibly *pyrG*89; possibly ∆*nkuA::argB*	This study
XX526	*hhoA*-GFP-*AfriboB*; ∆*capB*::*AfpyrG*; *pyrG*89; ∆*nkuA::argB; yA*2	This study

### Live cell imaging and analyses

Fluorescence microscopy of live *A*. *nidulans* hyphae was as described^28,29^. All microscopic images were captured using an Olympus IX70 inverted fluorescence microscope linked to a PCO/Cooke Corporation Sensicam QE cooled CCD camera. An UplanApo 100x objective lens (oil) with a 1.35 numerical aperture was used. A filter wheel system with GFP/mCherry-ET Sputtered series with high transmission (Biovision Technologies) was used. The IPLab software was used for image acquisition and analysis. For all images except for those presented in Fig. 6, cells were grown in the LabTek Chambered #1.0 borosilicate coverglass system (Nalge Nunc International, Rochester, NY) for ~8 hours or overnight at 32 °C or 37 °C and images were taken at room temperature. For the analysis on the frequency and velocity of early endosome transport (Fig. 6), cells were grown for overnight at 37 °C in DTC3 culture dishes (Bioptechs, Butler, PA), and time lapse sequences were captured at 37 °C by using a Bioptechs heating stage and heating objective system. All the images were taken with a 0.1-s exposure time (binning: 2 × 2). The IPLab image files were copy-pasted directly to Adobe Photoshop without any brightness adjustment. For studying the early endosome-distribution phenotype of the ∆*capA* mutant, the hyphal-tip region was defined as a region within ~2 μm to the hyphal tip. For measuring early endosome movement, we focused on movements within ~7 μm to the hyphal tip within the hyphal-tip cell (“hyphal-tip cell” refers to the hyphal segment between the hyphal tip and the most proximal septum). Retrograde movements initiating from the hyphal tip region are defined as “dynein-mediated”, and tip-directed movements are defined as “kinesin-mediated”. 30 frames were taken for each sequence with a 0.1-s exposure time and a 0.3-s interval between frames, and the “generate-kymograph” and “measure-kymograph functions” of IPLab were used for analysis.

### Construction of the strain containing the *capA*-GFP allele at the *capA* locus

For constructing the CapA-GFP fusion, we first used the following oligos to amplify CapA genomic DNA and the GFP-*AfpyrG* fusion from the plasmid pFNO3 (deposited in the FGSC by Steve Osmani)^73,74^: U1C (5′-CCGCCCCTTTCCACCAGAGATATCC-3′), U1N (5′-GTAACGGCAGGTCATTGTGTCCGAA-3′), D2N: 5′-TAGGCTGGACCTCAGTGGTTTTTCT-3′), D2C (5′-TGCTATAGCGTTACCTTTACGACCA-3′), GFPy5 (5′-GGATATCTCTGGTGGAAAGGGGCGGGGAGCTGGTGCAGGCGCTGGA-3′) and GFPy3 (5′-AGAAAAACCACTGAGGTCCAGCCTACTGTCTGAGAGGAGGCACTGAT-3′). Specifically, U1C and U1N were used to amplify the 1-kb fragment in the codon region, and D2N and D2C were used to amplify the 1-kb fragment in the 3′ untranslated region, using wild-type genomic DNA as template, and GFPy5 and GFPy3 were used to amplify the 2.7-kb GFP-*AfpyrG* fragment using the pFNO3 plasmid DNA as template. We then used two oligos, U1N and D2C, for a fusion PCR of the three fragments to generate the CapA-GFP-Af*pyrG* fragment that we used to transform into a wild-type strain containing **∆***nkuA*^75^. The transformants were screened by microscopically observing the GFP signals at actin patches near the hyphal tip, and the homologous integration of the fusion DNA into the endogenous *capA* locus was confirmed by PCR. Specially, a 1.1-kb product was obtained with the two following oligos: AfpyrG (5′-AGCAAAGTGGACTGATAGC-3′) and CapACR (5′-AACGACCCAGAGAAGACAG-3′).

### Construction  of  the  ∆*capA*  and  ∆*capB*  mutants

For constructing the ∆*capA* mutant, we first made the ∆*capA* construct with the selective marker *pyrG* from *Aspergillus fumigatus*, *AfpyrG*, in the middle of the linear construct^72^. Specifically, we used CAPAPG5 (5′-CCTGTGACATGATCAATCGGGTTCGTGCTCTTCTCCCTCTTCGCG-3′) and CAPAPG3 (5′-AAAACCACTGAGGTCCAGCCTACCGCCCCTTTCTGTCTGAGAGGAGGCACTG-3′) as primers and the pFNO3 plasmid (deposited in the FGSC by Steve Osmani)^73,74^ as template to amplify a 1.9-kb *AfpyrG* fragment. We used CAPAd5 (5′-AGTGCAAGGAAGGATTCAACCACTAGCGC-3′) and CAPANN3 (5′-CGAACCCGATTGATCATGTCACAGG-3′) as primers and wild-type genomic DNA as template to amplify a 1-kb fragment upstream of CapA coding sequence. We used CAPADD5 (5′-AAAGGGGCGGTAGGCTGGACCTCAGTGGTTTT-3′) and CAPAd3 (5′-GCGTTACCTTTACGACCAATGTCTGTCCCACC-3′) as primers and wild-type genomic DNA as template to amplifying a 1-kb fragment downstream of the CapA coding sequence. We then performed a fusion PCR to fuse the three fragments and obtained a 3.9-kb fragment, which we transformed into the *A*. *nidulans* strain XY42 containing **∆***nkuA*^75^. Several transformants were obtained that show a “small-colony” phenotype. Homologous integration of the deletion construct was confirmed by PCR using the following two pairs of primers: CapAN5 (5′-GTGAGTGGAGTTTCGTAACCC-3′) and AFpyrG3 (5′-GTTGCCAGGTGAGGGTATTT-3′); CapACR (5′-AACGACCCAGAGAAGACAG-3′) and AFpyrG5 (5′-AGCAAAGTGGACTGATAGC-3′). Note that in this deletion strain, we have deleted the whole open reading frame plus ~420 bp 5′ UTR of the *capA* gene.

To further confirm that the “small-colony” phenotype is caused by the ∆*capA* mutant allele, we crossed the ∆*capA* mutant with the strain carrying the CapA-GFP fusion gene integrated at the *capA* locus. We analyzed 32 recombinant progenies (containing different nutritional markers from the parental strains) from this cross, of which 14 are small colonies, and 18 are of normal size. The 18 normal-sized colonies all contained CapA-GFP signals, but none of the 14 small colonies contained CapA-GFP signals. Because the *capA* locus in a haploid can only contain either the CapA-GFP allele or the ∆*capA* allele but not both, this result is exactly as expected if the “small-colony” phenotype is causally linked to the ∆*capA* allele.

For constructing the ∆*capB* mutant, we used a strategy similar to that used for making the ∆*capA* mutant but using six different primers: CAPBPG5 (5′-ACCACTATAATGGCGGACGCCCAATTGCTCTTCTCCCTCTTCGCG -3′), CAPBPG3 (5′- AAACTGTACACAGCCTACCGCTGCAGTCTGTCTGAGAGGAGGCACTG -3′), CAPBd5 (5′-CAGAATTTATCCGCTATCCTCGAAGCGACGC -3′), CAPBNN3 (5′- ATTGGGCGTCCGCCATTATAGTGGT-3′), CAPBDD5 (5′-ACTGCAGCGGTAGGCTGTGTACAGTTT-3′) and CAPBd3 (5′-CGATGACTGAAAGCAACGATTCTGGGG-3′) for obtaining the AfpyrG, 1 kb upstream of capB coding region and 1 kb downstream of capB coding region.

A fusion PCR was performed to fuse the above 3 fragments into a 3.9 -kb fragment (oligo: CAPBd3 and CAPBd5**)**, followed by transformation into the strain XY42. The deletion strains were selected by colony phenotype and confirmed by PCR using these two pairs of primers: CapBDN5, 5′-ACCAGAATGTAGGAGGCACT-3′ and AFpyrG3, 5′-GTTGCCAGGTGAGGGTATTT-3′; CapBDC3, 5′-ACATAAATCTAAGCAAGAAAATACG-3′and AFpyrG5, 5′-AGCAAAGTGGACTGATAGC-3′.

### Experiments showing co-segregation of the ∆*capA* or ∆*capB* mutation with a defect in nuclear distribution or early-endosome distribution

To study nuclear distribution in the ∆*capA* mutant, we crossed a ∆*capA* transformant (XY42 background) with the strain XX390 containing GFP-labeled histone H1. Because both XX42 and XX390 contain the *pyrG*89 mutation, and because the ∆*capA* allele is marked by the selective marker *AfpyrG* (*A*. *fumigatus pyrG*), only those progenies containing the ∆*capA* allele are expected to grow on plates lacking uridine and uracil. Indeed, only small colonies characteristic of the ∆*capA* mutant grew on a MM + glucose plate without supplements. 16 progenies were randomly selected and cultured in MM + glucose medium at 37 °C for ~8 hours for microscopic examination, and 9 of them were found to carry GFP-labeled histone H1. All of these 9 strains exhibited abnormal nuclear distribution as 2, 3 or more nuclei can often be observed in the spore head, a phenotype never observed in wild-type strains grown under the same conditions. This result suggests that the nuclear-distribution defect is linked to the ∆*capA* mutation.

To study early-endosome distribution in the ∆*capA* mutant, we crossed a ∆*capA* strain (JZ711) with the strain MAD3131 containing GFP-RabA driven by the *gpdA*^mini^ promoter. Because the MAD3131 strain contains the *gpdA*^mini^::GFP-RabA-linked *pyroA* selective marker compensating for its *pyroA*4 mutation, and because the JZ711 also contains the *pyroA*4 mutation, only the progeny containing GFP-RabA grew on a MM + glucose plate missing pyridoxine. We randomly selected 9 ∆*capA* and 8 wild-type progenies and cultured them in MM + glucose medium at 37 °C for ~8 hours for microscopic examination. While all of the wild-type strains showed normal early-endosome distribution, all of the ∆*capA* strains showed abnormal early-endosome distribution as the number of germ tubes containing the hyphal-tip-localized early endosome is abnormally high. This result suggests that the early endosome-distribution phenotype is linked to the ∆*capA* mutation.

Finally, we also crossed a ∆*capB* transformant (XY42 background) with XX390 and analyzed the progenies. As expected, small colonies characteristic of the ∆*capB* mutant were the only ones that grew on a MM + glucose plate (without supplements). 28 progenies were randomly selected and cultured in MM + glucose at 37 °C for ~8 hours for microscopic examination, and 14 of them were found to carry GFP-labeled histone H1. All of these 14 strains exhibited a defect in nuclear distribution as 2, 3 or more nuclei can often be observed in the spore head (Supplemental Fig. 1^3^), suggesting that the nuclear-distribution defect is linked to the ∆*capB* mutation. Since the *capA* and *capB* genes are located on different chromosomes, these results provide extremely strong support for the conclusion that the nuclear-distribution and early endosome-distribution defects are caused by loss of the capping protein.

### Construction of the strain containing the ∆C-HookA-S allele

We first made a strain containing the HookA-S allele, which is similar to what has been described for the construction of the HookA-GFP-containing strain^29^, except that the plasmid pAO81 instead of pFNO3 was used. We then used the genomic DNA from the HookA-S-containing strain as well as from the ∆C-HookA-GFP-containing strain^29^ as template for making the strain containing the ∆C-HookA-S allele. Specifically, HKORFF (5′-AAACGACGAGCAGCAGCTG-3′) and ∆C37R (5′-TGGACCAGCAACGGCACTTCTTTGTGAACTCATGAGGGCGAG-3′) were used to amplify a ~0.8 -kb fragment from the strain containing ∆C-HookA-GFP, and HKFusF (5′-AAGAAGTGCCGTTGCTGGTCCAGGAGCTGGTGCAGGCGCTGGAG-3′) and HKUTRR (5′-TAACTGTTGAAGGAGATCC-3′) were used to amplify a ~2.7 -kb fragment from the HookA-S strain, and the two fragments were fused together by fusion PCR using HKORFF and HKUTRR as primers. The product (~3.5 kb) was transformed into the RQ54 strain, and the phenotype of the ∆C-HookA-S-containing strain was verified by observing the mCherry-RabA fusion protein, which shows an abnormal buildup at the hyphal tip^29^, as well as by using the anti-S-tag antibody (Cell Signaling Technology, Inc.) for western analysis.

### Biochemical pull-down assays, western analysis and mass spectrometry analysis

The μMACS GFP-tagged protein isolation kit (Miltenyi Biotec) was used to pull down dynein and dynactin. This was done as described previously^29^. About 0.4 g hyphal mass was harvested from overnight culture for each sample, and cell extracts were prepared using a lysis buffer containing 50 mM Tris-HCl, pH 8.0 and 10 μg/mL of a protease inhibitor cocktail (Sigma-Aldrich). Cell extracts were centrifuged at 8,000 *g* for 15 minutes and then 16,000 *g* for 15 minutes at 4 °C, and supernatant was used for the pull-down experiment. To pull down GFP-tagged proteins, 25 μL anti-GFP MicroBeads were added into the cell extracts for each sample and incubated at 4 °C for 30 minutes. The MicroBeads/cell extracts mixture was then applied to the μColumn followed by gentle wash with the lysis buffer used above for protein extraction (Miltenyi Biotec). Pre-heated (95 °C) SDS-PAGE sample buffer was used as elution buffer. Western analyses were performed using the alkaline phosphatase system and blots were developed using the AP color development reagents from Bio-Rad. Quantitation of the protein band intensity was done using the IPLab software as described previously^76,77^. The antibody against GFP was from Clontech (polyclonal). The antibodies against dynein HC, dynactin p150 and Arp1 were described previously^6,40^. For proteomic analysis, eluted protein samples were run on an SDS-PAGE gel until proteins had reached the interphase between the stacking and separating gels as previously described^78^. A single gel slice containing the proteins was sliced out for mass spectrometry analysis of each sample, which was done using the Taplin Mass Spectrometry Facility at Harvard Medical School (two original data sets are provided as Supplemental dataset 1 and Supplemental dataset 2).

### Data Availability

All the relevant data generated during this study are included in this published article (and its Supplementary Information files). The datasets analyzed during the current study are available from the corresponding author on reasonable request.

## Electronic supplementary material

Supplemental infomation

supplementary data set 1

supplementary data set 2

Movie 1

Movie 2

Movie 3
