# Supplementary material for: The actin capping protein in Aspergillus nidulans enhances dynein function without significantly affecting Arp1 filament assembly
Source: Sci Rep. 2018 Jul 30;8:11419. doi: 10.1038/s41598-018-29818-4 (PMC6065395; doi:10.1038/s41598-018-29818-4)
Supplement: Supplementary file 1 — Supplemental infomation [file 41598_2018_29818_MOESM1_ESM.pdf]

Supplementary information

The actin capping protein in *Aspergillus nidulans* enhances dynein function without significantly affecting Arp1 filament assembly

Jun Zhang, Rongde Qiu and Xin Xiang*

Department of Biochemistry and Molecular Biology, The Uniformed Services University of the Health Sciences- F. Edward Hébert School of Medicine, Bethesda, Maryland 20814, USA.

*Corresponding author

Tel: (301) 295-0000
Fax: (301) 295-9598
Email: xin.xiang@usuhs.edu

This supplementary information file contains 13 Supplemental figures and 1 Supplemental table.

Two Supplementary Datasets containing the original Mass Spectrometry data are provided to journal website as excel files. Supplementary Dataset 1 corresponds to Table 1. Supplementary Dataset 2 corresponds to Supplemental Table 1 (but with an extra sample of CapA-GFP/Δ*hookA*). Sample names are labeled in red on top of the excel files.

Three Supplementary movies (linked to Figure 6A) are provided.

Movie 1 TagGFP2-RabA-labeled early endosomes in a wild-type strain. Cells were grown for overnight at 37°C, and time lapse sequences were captured at 37°C. 30 frames were taken with a 0.1-s exposure time and a 0.3-s interval between frames. Binning: 2x2. The movie speed has been increased 10-fold.

Movie 2 TagGFP2-RabA-labeled early endosomes in the Δp25 mutant. Cells were grown for overnight at 37°C, and time lapse sequences were captured at 37°C. 30 frames were taken with a 0.1-s exposure time and a 0.3-s interval between frames. Binning: 2x2. The movie speed has been increased 10-fold.

Movie 3 TagGFP2-RabA-labeled early endosomes in the Δ*capA* mutant. Cells were grown for overnight at 37°C, and time lapse sequences were captured at 37°C. 30 frames were taken with a 0.1-s exposure time and a 0.3-s interval between frames. Binning: 2x2. The movie speed has been increased 10-fold.

Supplemental Figure 1. A sequence alignment of *A. nidulans* CapA (A.n.) with human (H.s.) and yeast (S.c.) capping protein alpha. The alignment was done using CLUSTALW (http://npsa-pbil.ibcp.fr/cgi-bin/npsa_automat.pl?page=/NPSA/npsa_clustalw.html). Residues that are identical (*), strongly similar (:) or weakly similar (.) are shown as red, green and blue characters respectively.

```
A.n.      -------MAST--VEFASSFIEGAPPGELADVVSDIKTLTSDGDDIIPSLAPAFERYNES 51
H.s.      MADLEEQLSDEEKVRIAAKFIIHAPPGEFNEVFNDVRLLLNNDNLLREGAAHAFAQYNLD 60
S.c.      -------MSSSKFEEVINKIINDSPPGELREVYDDLIKITSENS--KNTILDAIENYNVQ 51
                 ::.    ..  .:*  :****: :* .*:  :  .:..        *: .** .

A.n.      QLATVKLPGASQEVIVSEFNRLEGSRYFDVESQTSFEVDHITQSTSAAQSYVLESQNAD- 110
H.s.      QFTPVKIEGYEDQVLITEHGDLGNGKFLDPKNRICFKFDHLRKEATDPRPCEVENAVES- 119
S.c.      NCIPIEVNGNS--VIISKYN-KEGAKFFDPVNSVIFSVNHLERKGLDIEPYEFTHAKIEK 108
          :  .::: * .  *::::..   ..:::*  .   *..:*: :.    ..  .     .

A.n.      -LIKSLLKTLGAHAREHYP-SSSYGVYPIEKDS--AIAILLVANRYSPNNFWNGRYRSIY 166
H.s.      -WRTSVETALRAYVKEHYP-NGVCTVYGKKIDGQQTIIACIESHQFQAKNFWNGRWRSEW 177
S.c.      GQLKELHDKLHEYLLQSFPGDVSFAVYPVPEEIS-KISIIIVSTKYNPNNFWNGHWRSSY 167
             ..:   *  :  : :* .    **    :    *   : : ::..:*****::** :

A.n.      QFPVG-DSTTITGKIHVDVHYYEDGNVALNTTKPLNISVPN----ASAESIISRIASAER 221
H.s.      KFTITPSTTQVVGILKIQVHYYEDGNVQLVSHKDIQDSLTVSNEVQTAKEFIKIVEAAEN 237
S.c.      IYDL--ETRELSGQISTQVHYYEDGNVSFQSGKDINQ--------SNVDDVVCTIRDIET 217
           : :  .:  : * :  :********* : : * ::          .....:  :   *

A.n.      NYQEELNKAFGQMAEGAFKSLRRQLPITRQKVEW-EKVGGYRLGQDISGGKGR 273
H.s.      EYQTAISENYQTMSDTTFKALRRQLPVTRTKIDW-NKILSYKIGKEMQNA--- 286
S.c.      NFENDLDLSFFDLNEKQFKALRRRLPVTRSKINWGSAIGSYRLGKNAAEGK-- 268
          :::  :.  :  : :  **:***:**:** *::* . : .*::*::   .
```

Supplemental Figure 2. A sequence alignment of *A. nidulans* CapB (A.n.) with human (H.s.) and yeast (S.c.) capping protein beta. The alignment was done using CLUSTALW (http://npsa-pbil.ibcp.fr/cgi-bin/npsa_automat.pl?page=/NPSA/npsa_clustalw.html). Residues that are identical (*), strongly similar (:) or weakly similar (.) are shown as red, green and blue characters respectively.

```
A.n.      MADAQFDSALDLLRRLNPRDTKQNLQAITSIVPDLTEDLLSSVDQPLEIRRCPK-TKRDY 59
H.s.      MSDQQLDCALDLMRRLPPQQIEKNLSDLIDLVPSLCEDLLSSVDQPLKIARDKV-VGKDY 59
S.c.      MSDAQFDAALDLLRRLNPTTLQENLNNLIELQPNLAQDLLSSVDVPLSTQKDSADSNREY 60
          *:* *:*.****:*** *   ::**. : .: *.* :******* **.  :      ::*

A.n.      LLCDYNRDGDSYRSPWSNEFDPPL-----DDGTVPSERVRRLEVAANEAFDVYRELYYEG 114
H.s.      LLCDYNRDGDSYRSPWSNKYDPPL-----EDGAMPSARLRKLEVEANNAFDQYRDLYFEG 114
S.c.      LCCDYNRDIDSFRSPWSNTYYPELSPKDLQDSPFPSAPLRKLEILANDSFDVYRDLYYEG 120
          * ****** **:****** : * *     :*...**  :*:**: **::** **:**:**

A.n.      GVGSVYFWDLD------DGFAGVILLKKGVSPGGKHSGEWDSIHVFEATDR---GRMAHY 165
H.s.      GVSSVYLWDLD------HGFAGVILIKKAGDGSKKIKGCWDSIHVVEVQEKSS-GRTAHY 167
S.c.      GISSVYLWDLNEEDFNGHDFAGVVLFKKNQSD----HSNWDSIHVFEVTTSPSSPDSFNY 176
          *:.***:***:      ..****:*:**  .      . ******.*.          :*

A.n.      KLTSTVILHLSNENEAL-GEMDLSGNMTRQIEVDMNVDSD-------ASHVANVGKLVED 217
H.s.      KLTSTVMLWLQTNKSGS-GTMNLGGSLTRQMEKDETVSDC-------SPHIANIGRLVED 219
S.c.      RVTTTIILHLDKTKTDQNSHMMLSGNLTRQTEKDIAIDMSRPLDVIFTSHVANLGSLIED 236
          ::*:*::* *.. :    . * *.*.:*** * *  :.         :.*:**:* *:**

A.n.      MELKMRNLLQEVYFGKAKDVVGELRSIGPLSETNRD-RATHQEMIRGLQR---- 266
H.s.      MENKIRSTLNEIYFGKTKDIVNGLRSVQTFADKSKQ-EALKNDLVEALKRKQQC 272
S.c.      IESQMRNLLETVYFEKTRDIFHQTKNAAIASSAEEANKDAQAEVIRGLQSL--- 287
          :* ::*. *: :** *::*:.         :. ..  .  : :::..*:
```

Supplemental Figure 3. Colony phenotype of the Δ*capA* and Δ*capB* mutants in comparison to their wild type parental strain (yellow), and colony phenotype of the CapA-GFP strain in comparison to its wild type parental strain (green). The plate was incubated at 37°C for 2 days.

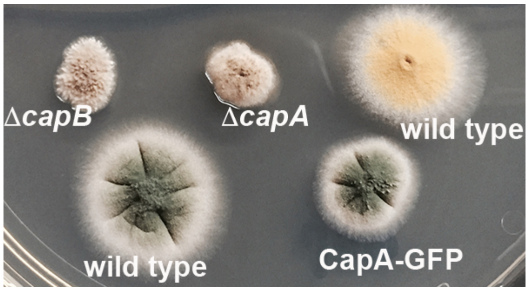

Supplemental Figure 4. Localization of CapA-GFP and AbpA-mRFP[1] signals. Merged images were shown on the right, indicating co-localization of the CapA-GFP and AbpA-mRFP signals. Bar, 5 μm.

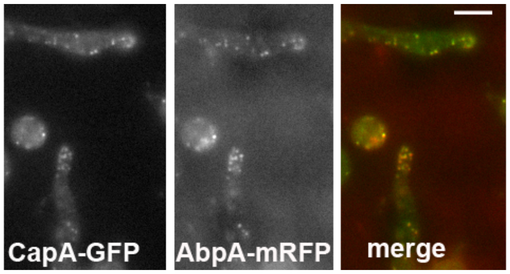

Supplemental Figure 5. This figure contains the original western blots for Figure 1B, which shows that dynactin p150, Arp1 and the dynein HC were pulled down with CapA-GFP. A strain without any GFP tag was used as a negative control. After protein gel transfer, the single nitrocellulose membrane was cut into several pieces to be probed by several antibodies that recognize proteins of different sizes. The antibody against GFP (from Clontech) has been used previously[2]. The affinity-purified antibodies against dynein HC, dynactin p150 and Arp1 have been described and used previously[3,4].

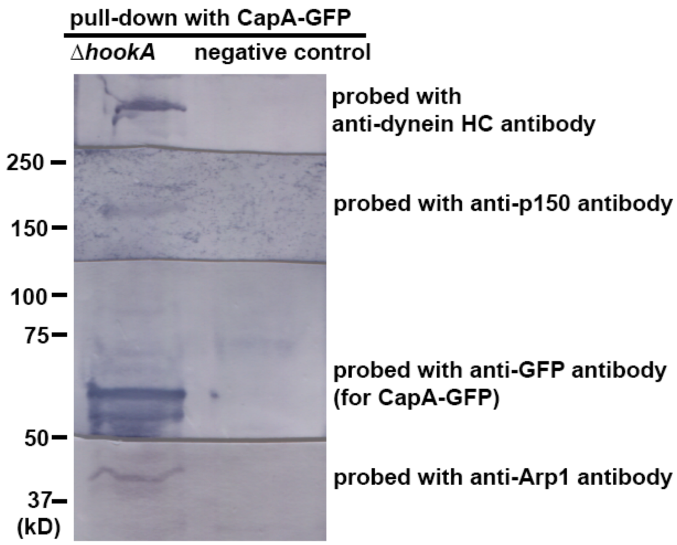

Supplemental Figure 6. Site-specific integration of the Δ*capA* construct into the genome. (A) A diagram showing the Δ*capA* linear construct with the *AfpyrG* marker flanked by the 5' and 3' flanking sequences of the *capA* gene. Homologous recombination events occurred between this construct and the wild-type genome (wild type) are indicated by crosses. The resulting Δ*capA* locus is shown at the bottom. The positions of the primers used for PCR analyses are indicated by arrows. Note that the primers CapAN5 and CapACR are located outside of the flanking sequences of the Δ*capA* construct and thus the PCR reactions will produce the expected products of 1.05 and 1.1 kb only when the construct integrated into the *capA* locus. (B) Result of a PCR analysis on genomic DNAs from the Δ*capA* mutant and a wild type strain. Picture of a Ethidium Bromide-stained DNA agarose gel is shown, and primers are indicated on the right.

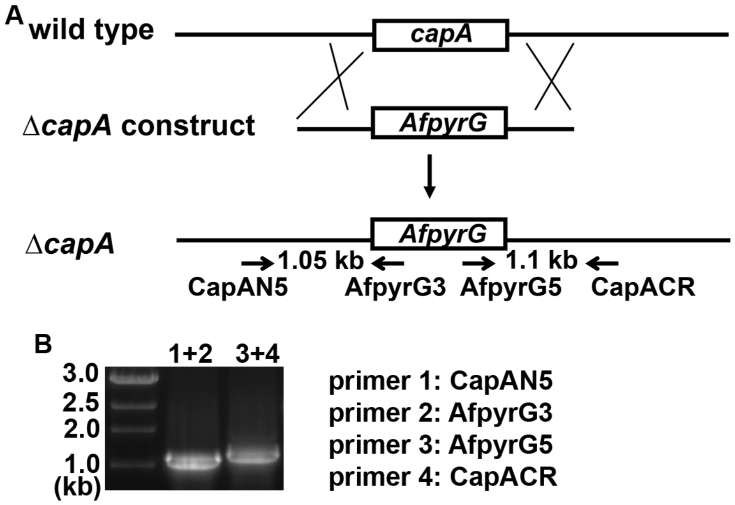

Supplemental Figure 7. Quantitation on nuclear distribution in wild type and the Δ*capA* mutant. (A) Percent of wild-type (n=91) and Δ*capA* (n=119) germ tubes showing different numbers of nuclei in the spore head. Cells were cultured overnight at 32°C in MM+glycerol medium. The mean ranks of these two sets of data are significantly different at the *p*-value of 0.0001 (p=0.000000000390825, two-tailed) based on a nonparametric test without assuming Gaussian distribution (unpaired, Mann-Whitney test). (B) Percent of wild-type (n=73) and Δ*capA* (n=58) germ tubes showing different numbers of nuclei in the spore head. Cells were cultured overnight at 32°C in MM+glucose medium. The mean ranks of these two sets of data are significantly different at the *p*-value of 0.0001 (p=0.000010504657003, two-tailed) based on a nonparametric test without assuming Gaussian distribution (unpaired, Mann-Whitney test). (C) Percent of wild-type (n=111) and Δ*capA* (n=118) germ tubes showing different numbers of nuclei in the spore head. Cells were cultured overnight at 37°C in MM+glycerol medium. The mean ranks of these two sets of data are significantly different at the *p*-value of 0.0001 (p<0.000000000000001, two-tailed) based on a nonparametric test without assuming Gaussian distribution (unpaired, Mann-Whitney test). All the nonparametric tests were done by using Prism 7.

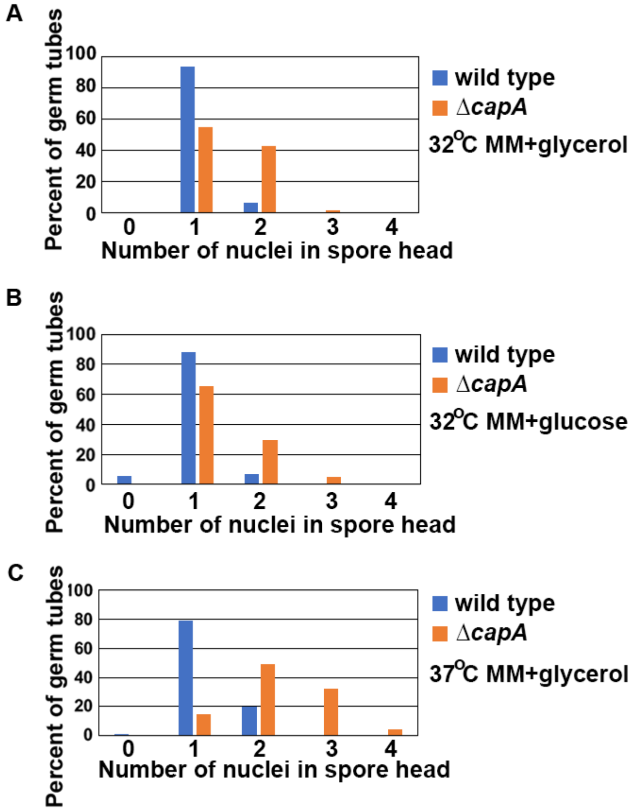

Supplemental Figure 8. Quantitation on the distribution of early endosomes labeled by *gpdA*^{mini}-driven GFP-RabA in wild type, the Δ*capA* mutant and the Δp25 mutant. (A) Percent of wild type (n=102), Δ*capA* (n=160) and Δp25 (n=100) germ tubes showing hyphal-tip localization of an early endosome after cells were cultured overnight at 32°C in MM+glycerol medium. The percentage values are shown on the graph with error bars representing the confidence interval (95% confidence) values generated by Prism 7 for Mac OS X (version 7.0c, 2017). (B) Percent of wild type (n=139), Δ*capA* (n=165) and Δp25 (n=50) germ tubes showing hyphal-tip localization of an early endosome after the cells were cultured at 37°C in MM+glycerol medium. The percentage values are shown on the graph with error bars representing the 95% confidence interval values generated by Prism 7.

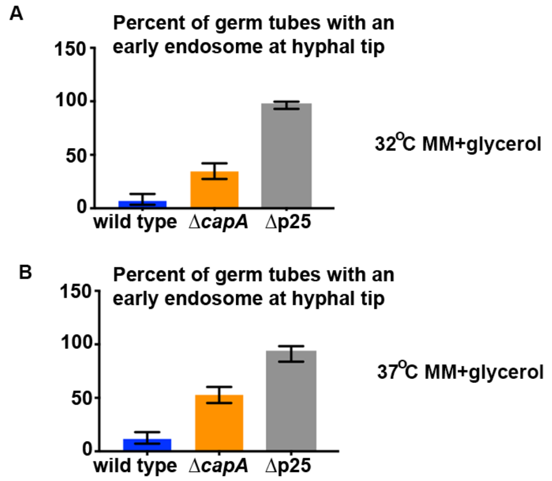

Supplemental Figure 9. Localization of GFP-dynein HC and p150-GFP signals in wild-type and Δ*capA* cells. Cells were cultured for ~8 hours at 37°C in MM+glucose medium. Bar, 5 μm.

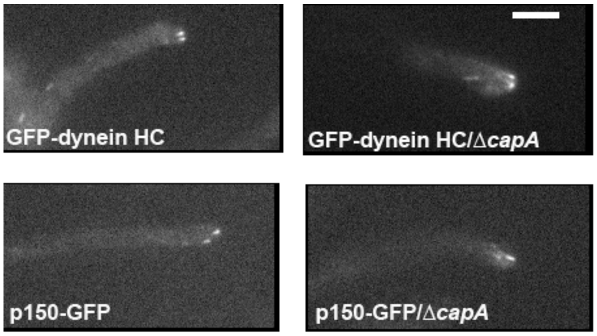

Supplemental Figure 10. This figure contains the original western blots for Figure 5A, which shows that apparently normal amounts of dynein HC and Arp1 are pulled down with p150-GFP in the Δ*capA* mutant extract. The leftmost lane was not used in Figure 5A because the result is similar to that shown in Figure 1B. A strain without any GFP tag was used as a negative control. Another control (on the right) is the strain containing GFP-dynein HC in the *alcA*-Arp1 background, in which the expression of Arp1 was repressed by glucose, which affects the stability of p150[4]. The antibody against GFP (from Clontech) has been used previously[2]. The affinity-purified antibodies against dynein HC, dynactin p150 and Arp1 have been described and used previously[3,4].

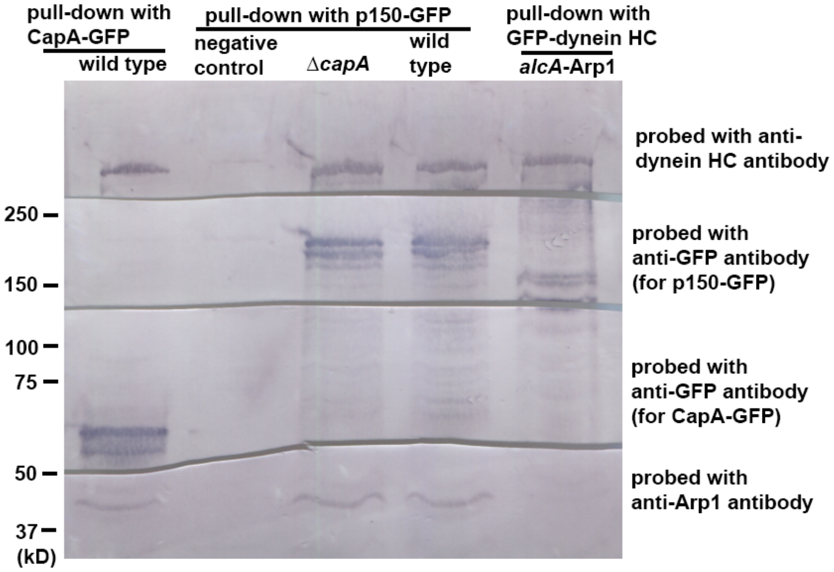

Supplemental Figure 11. This figure contains the original western blots for Figure 5B (flipped horizontally), which shows that dynein HC, dynactin p150 and Arp1 are pulled down with ΔC-HookA-GFP in the Δ*capA* mutant extract. The antibody against GFP (from Clontech) has been used previously[2]. The affinity-purified antibodies against dynein HC, dynactin p150 and Arp1 have been described and used previously[3,4].

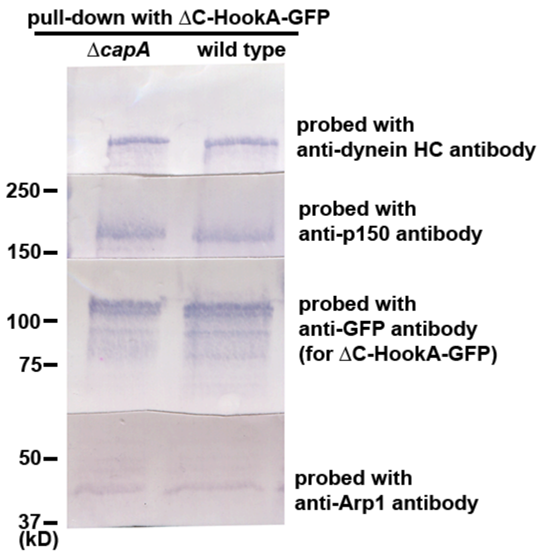

Supplemental Figure 12. Dynein and dynactin interact with HookA-GFP in the Δ*capA* mutant but not in the Δp25 mutant. (A) Western blots showing that dynein HC and p150-GFP are pulled down with HookA-GFP normally in the Δ*capA* mutant but not in the Δp25 mutant. (B) Original western blots for (A). The brightness of the whole upper blot was reduced so that the dynein HC bands can be seen more easily. The antibody against GFP (from Clontech) has been used previously[2]. The affinity-purified antibodies against dynein HC and dynactin p150 have been described and used previously[3,4].

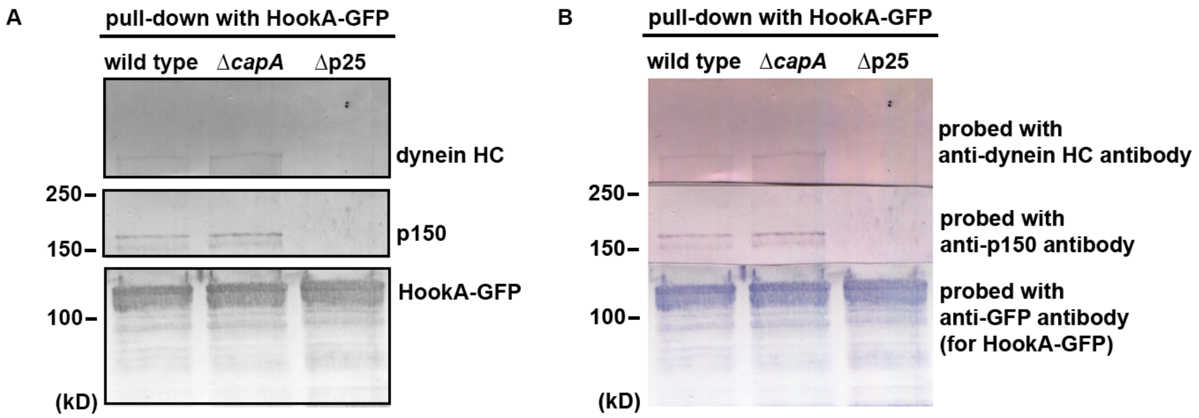

Supplemental Figure 13. The nuclear-distribution phenotype of the Δ*capB* mutant. (A) Cells were grown at 37°C for ~8 hours in MM+glucose medium. A wild-type strain was used as a control. (B) Cells were grown at 37°C for overnight in MM+glycerol medium. Both wild type and the Δ*capA* mutant were used as controls. Bar, 5 μm.

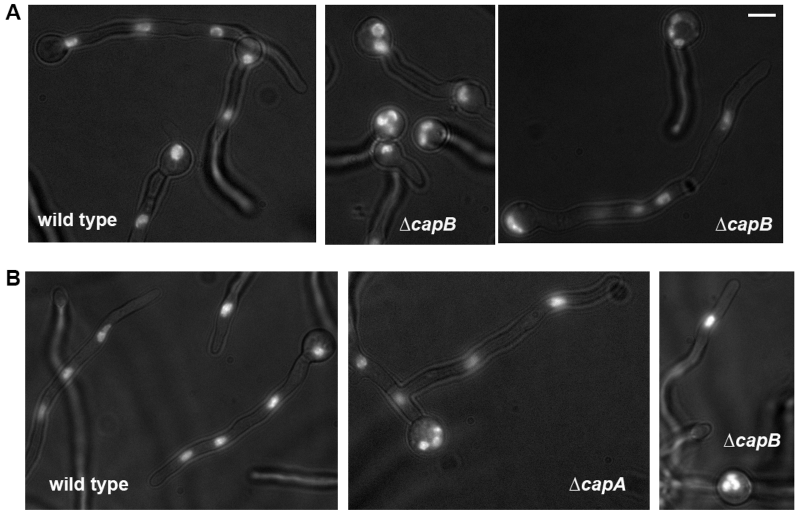

Supplemental Table 1 Proteomic analysis of proteins pulled down with p150-GFP in wild type or the ∆*capA* background. Protein names and the number of unique peptides detected are listed. Protein extracts of the two strains are processed the same way in the experiment (see Supplementary Dataset 2 for the original data).

	p150-GFP	p150-GFP/∆*capA*
CapA (An2126, 273aa)	5	0
CapB (An0290, 266aa)	4	0
Arp1 (An1953, 380aa)	8	11
Arp11 (An3185, 557aa)	10	9
p62 (An4917, 637aa)	4	3
p25 (An5022, 202aa)	4	4
p150 (An6323, 1342aa)	54	51
p50 (An3589, 467aa)	12	17
p24 (An12001, 235aa)	1	2
Dynein HC (An0118, 4345aa)	89	69

References

1 Araujo-Bazan, L., Penalva, M. A. & Espeso, E. A. Preferential localization of the endocytic internalization machinery to hyphal tips underlies polarization of the actin cytoskeleton in Aspergillus nidulans. *Molecular microbiology* **67**, 891-905, doi:10.1111/j.1365-2958.2007.06102.x (2008).

2 Zhang, J., Qiu, R., Arst, H. N., Jr., Penalva, M. A. & Xiang, X. HookA is a novel dynein-early endosome linker critical for cargo movement in vivo. *J Cell Biol* **204**, 1009-1026, doi:10.1083/jcb.201308009 (2014).

3 Xiang, X., Roghi, C. & Morris, N. R. Characterization and localization of the cytoplasmic dynein heavy chain in Aspergillus nidulans. *Proc Natl Acad Sci U S A* **92**, 9890-9894 (1995).

4 Zhang, J. *et al.* Arp11 affects dynein-dynactin interaction and is essential for dynein function in Aspergillus nidulans. *Traffic* **9**, 1073-1087, doi:10.1111/j.1600-0854.2008.00748.x (2008).
